# Letter to editor; delayed ophthalmotoxic effects of sulfur mustard and clean up the contaminated soils

**DOI:** 10.1186/2008-2231-21-20

**Published:** 2013-03-11

**Authors:** Seyed mansour Razavi, Payman Slalamati

**Affiliations:** 1 Department of community medicine, Tehran University of Medical Sciences, Tehran, Iran; 2 Sina Trauma and Surgery Research Center, Tehran University of Medical Sciences, Tehran, Iran

## Dear Editor-in-Chief

With great thanks, we noted the reasonable suggestions presented in the recent commentary [1] about our article on toxicity of mustard [2]. It was commented that the frequency of ophthalmic complications due to sulfur mustard should be 39.3% based on the cited study [3] while it was reported 93.3%. Notably, the frequencies of these complications were reported 65% [4] and 94% [5] in other studies that bring up a challenging issue. Therefore, we appreciate Shadboorestan’s attention and notice that a typesetting error has occurred, and the correct number is 39.3%.

Furthermore, in the commentary, concerns regarding the need for decontamination of the soil around the battle fields were come up [1]. We had already mentioned it in our article by terms of "building healthy soil". We meant that the lands should be cleaned and prepared not only for tourism purposes but also for re-habitation and agricultural activities. Low solubility of sulfur mustard in water (920 mg/L) and ease of hydrolysis once dissolved prevents from transport of mustard through the soil into the groundwater [6], and thus it remains in the soil. Therefore, decontamination is necessary we believe. After the chemical attacks in Halabja (a Kurdish town in Iraq) by that time Iraqi army, the farmers and inhabitants were withdrawn from cultivating and inhabiting in their villages. After finishing the war, most of the farmers returned to their contaminated villages and farmlands. Eighteen months later, some people complained ill-growth of plants in their farmlands. When the soil samples were analyzed equal to five-year post mustard exposure, no residual hazard was recognized. This supports that harmful effects of mustard in such climatic conditions disappear about four to five-year post exposure by spontaneous hydrolysis [7]. Sulfur mustard breaks down into fewer toxic degradation products such as thiodiglycol, hydrochloric acid and sulfonium salts [8]. On the other hand, some fungi (e.g., basidiomycetes) are able to break the bond between the carbon and sulfur and hydrolytic de-chlorination lead to degradation of sulfur mustard in the soil [6]. In addition, it probably does not go through the vascular system of the plants [8], and it is improbable to remain in the soil for long years. Since the mustard is rapidly hydrolyzed in contact with water [9] and regarding the frequent rains in the southern part of Iran, it is unlikely to find any trace of mustard in the region.

## References

[B1] Shadboorestan A Commentary on: a review on delayed toxic effects of sulfur mustard in Iranian veterans DARU 2012 20 99 P:3 23351282 10.1186/2008-2231-20-99 PMC3584943

[B2] Razavi SM Salamati P Saghafinia M Abdollahi M A review on delayed toxic effects of sulfur mustard in Iranian veterans DARU 2012 20 51 2 8 23351810 10.1186/2008-2231-20-51 PMC3555992

[B3] Khateri S Ghanei M Keshavarz S Soroush M Haines D Incidence of lung, eye, and skin lesions as late complications in 34,000 Iranians with wartime exposure to mustard agent J Occup Environ Med 2003 45 1136 1143 10.1097/01.jom.0000094993.20914.d1 14610394

[B4] Balali-Mood M Hefazi M The clinical toxicology of sulfur mustard Archives of Iranian Medicine 2005 8 3 162 179

[B5] Holisaz MT Raigany SM Hafezy R Bakhshandeh H The role of chemical warfare agents in inducing peripheral neuropathy Kowsar Med J 2003 8 39 46

[B6] Agency for Toxic Substances and Disease Registry (ATSDR) Toxicological profile for sulfur mustard http://www.osha.gov/SLTC/sulfurmustard/index.html 38412219

[B7] Dlawer AA Ala' A Long term hazards of chemical weapon agents: analysis of soil samples from Kurdistan years after exposure to sulfur mustard and nerve agents 2005 http://www.dlawer.net/?q=node/71

[B8] US Army Chemical Materials Agency Fact sheet-sulfur mustard Bis (2-chloroethyl) sulfidehttp://www.pmcd.apgea.army.mil/fndocumentviewer.aspx?docid=003676688

[B9] Ashmore MH Nathanail CP A critical evaluation of the implications for risk based land management of the environmental chemistry of sulfur mustard Environ Int 2008 34 1192 1203 10.1016/j.envint.2008.03.012 18486211

